# Impact of Self-Efficacy Strategies Education on Self-Care Behaviors among Heart Failure Patients

**Published:** 2020-01

**Authors:** Nooshin Peyman, Fateme Shahedi, Mahbubeh Abdollahi, Hassan Doosti, Zohre Zadehahmad

**Affiliations:** 1 *Department of Health Education and Health Promotion, School of Health, Mashhad University of Medical Sciences, Mashhad, Iran.*; 2 *Department of Radiology, School of Paramedical Sciences, Torbat Heydariyeh University of Medical Sciences, Torbat Heydariyeh, Iran.*; 3 *Health Sciences Research Center, Torbat Heydariyeh University of Medical Sciences, Torbat Heydariyeh, Iran.*; 4 *Department of Public Health, School of Health, Torbat Heydariyeh University of Medical Sciences, Torbat Heydariyeh, Iran.*; 5 *Health Science Research Center, Mashhad University of Medical Sciences, Mashhad, Iran.*

**Keywords:** *Selfefficacy*, *Self care*, *Heart failure*

## Abstract

**Background: **Self-efficacy in self-care behaviors is an effective framework for measuring patients’ degree of ability to perform self-care behaviors that significantly affect their recovery process and quality of life. This study was designed to investigate the effects of education based on self-efficacy strategies on self-care behaviors in heart failure patients.

**Methods**: A semi-experimental study was conducted on 80 heart failure patients divided into 2 equal groups of test and control. The intervention group received three 60-minute practical and theoretical training sessions based on self-care and self-efficacy strategies, while the control group received the usual care services. Self-care behaviors and self-efficacy were evaluated before training, shortly after training, and 3 months after the training program using the Sullivan self-efficacy questionnaire for heart failure patients and the European self-care behaviors questionnaires.

**Results**: The mean age of the patients was 55.00±8.48 and 51.61±8.51 years in the intervention and control groups, respectively. Women comprised 73.7% (n=59) of the study population. The mean score for the self-care and self-efficacy questionnaires in the experimental group was 23.50±6.58 and 18.57±6.64, correspondingly, before the intervention, which increased to 42.64±6.74 (P<0.014) and 32.29±7.06 (P<0.001), respectively, shortly after the intervention. A significant improvement also occurred at 3 months’ follow-up (P<0.001). Self-care behaviors also revealed a positive correlation with self-efficacy shortly after the intervention (r=0.82, P<0.001) and 3 months after the intervention (r=0.85, P<0.001).

**Conclusion**: The implementation of educational interventions based on self-efficacy strategies could have positive effects on health-promoting behaviors among heart failure patients.

## Introduction

Heart failure (HF) is one of the most common cardiovascular disorders in both developed and developing countries and is considered to be a chronic, progressive, and debilitating disease.^     1 ^ Appropriate pharmacological measures, mechanical heart devices, and behavioral management can improve quality of life, life expectancy, and morbidity in patients with HF.^   2 ^ Because of the complexity of HF management, effective self-care among HF patients is crucial to the prevention of patients’ poor outcomes.^   3 ^ Self-care refers to activities and practices performed primarily by individuals in order to maintain and enhance health and overall well-being.^   4 ^ Based on the Riegel et al. model, self-care in HF patients signifies the methods in which patients actively participate in their own care and the management of their chronic condition.^   5 ^ In the abovementioned model, self-care, in the dimension of self-maintenance, reflects behaviors aimed at maintaining physiological stability, monitoring signs and symptoms, and complying with the prescribed treatments. Self-care management encompasses all actions on the part of patients to recognize and interpret their own symptoms and manage their own health.^   6 ^ Studies have shown that patients with low literacy and limited health knowledge use fewer disease prevention services,^   7 ^ do not have adequate follow-up care, and have inadequate self-care behaviors.^   8 ^ Therefore, these patients may use more health care services, which increases the rate of hospitalization and, consequently, imposes high medical costs on the health care system each year.^   9 ^ Human behavior theories are essential not only to the description and prediction of health behaviors but also to the design of an effective intervention aimed at enhancing healthy lifestyle behaviors. According to studies, one of the most famous theories on how to predict and describe behavior is the Bandura social learning theory.^   10 ^ It is one of the most widely used behavior change theories and is a cognitive process that focuses on the role of individuals’ self-confidence in their abilities. Self-efficacy is the most important construct of the Bandura social cognitive theory that refers to individuals’ belief in their capability to perform a particular task.^   10 ^ A person with low self-efficacy is less likely to attempt a new health behavior or change a behavior. Indeed, there is a significant association between self-efficacy, health behavior change, and effective health promotion.^   11 ^ In this research, the self-efficacy theory was applied in educational planning, as an important prerequisite for behavioral change,^     12 ^ and subsequently, the effects of a self-efficacy theory-based educational training program on promoting self-care behaviors were evaluated among HF patients.

## Methods

A semi-experimental study was conducted on HF patients admitted to the Cardiology Ward of Shahid Modarres Hospital of Kashmar, Iran, between February and September of 2016. The criteria for entering the study were comprised of the diagnosis of HF with a reduced left ventricular ejection fraction (≤35%) confirmed by a cardiologist, age ≥18 years, hospitalization for HF, and reading and writing literacy. Additionally, the inclusion criteria for the family members were having an effective role in patient care and having a consanguinity or affinity relationship with the patient. The exclusion criteria consisted of clinical deterioration in the patient’s condition and refusal to give consent for participation in the study. 

First, 250 questionnaires were distributed among the HF patients admitted to the cardiology wards, and ultimately 80 individuals willing to participate in the study were selected as the sample size. All the study participants were assigned to 2 equal groups of intervention and control. The intervention group was equally divided into 2 subgroups, who received three 60-minute training sessions within a month based on health literacy strategies. The control group received routine care services without receiving any education program by the health educators. All the participants received advice about the research tool and signed the informed consent form. In order to achieve the research goals, we collected data using demographic information questions, the Sullivan 5-level Likert *scale* self-efficacy questionnaire for cardiac patients, and the European self-care behaviors questionnaire. 

Both the control and intervention groups were assessed at 3 time points: before the intervention, shortly after the intervention, and at 3 months’ follow-up. In the current study, all the questionnaires were translated and culturally adapted to the Persian version using the Beaton protocol for *cross-cultural adaptation.*^13^ In this research, the average content validity index for the Sullivan self-efficacy questionnaire and the European self-care behaviors questionnaire was determined to be 88% and 85.3%, respectively. Furthermore, the reliability coefficient (Cronbach’s alpha) of the Sullivan self-efficacy questionnaire and the European self-care behaviors questionnaire was 85% and 89%, respectively, indicating the *good internal consistency* *of the test*.

During the first interview, sociodemographic information including age, gender, educational level, occupation, and place of residence was collected through personal interviews.

The Sullivan self-efficacy questionnaire consists of 13 questions used to measure patients’ confidence in observing public health, controlling symptoms, and complying with medication. The total score of the questionnaire is between 0 and 52, with higher scores representing better-perceived self-efficacy. 

The European self-care behaviors scale is a patient-reported questionnaire to measure self-care behaviors in patients with congestive HF. It consists of twelve 5-point Likert scale questions (Always = 5, and Never =1). The total score of this scale ranges from 12 to 60, and lower scores indicate poor observance of self-care behaviors by patients. 

After the completion of the questionnaires, the self-efficacy-based educational intervention was performed based on the focus-group discussion and three 60-minute weekly theoretical and practical educational sessions were held within 1 month. In the first session of the focus group discussion, an understanding of the importance of the disease, identification and control of HF signs and symptoms, and effective communication and participation of the group members in achieving the small preset goals were considered the larger goals (higher patient self-efficacy). In the training sessions, step-by-step performance of self-care behaviors was emphasized, and the patients were asked to express success in the next sessions, even if they were small (self-efficacy: successful experiences). The second session of the focus groups was conducted to train management and disease control (self-efficacy: succession experiences). At the end of the session, 2 patients who had successfully followed the recommendations were asked to share their experiences with the other participants in order to promote self-esteem and self-confidence in performing self-care behaviors and having a normal life. In the last session of focus group discussion, the physiological states resulting from the individuals’ self-assessment of their physical and psychological effects were investigated. Finally, the patients who did well in self-care behaviors were praised and verbal feedback was provided.

Data analysis was performed using Statistical Package for the Social Sciences (SPSS) software (SPSS for Windows, Version 16.0. Chicago, SPSS Inc.). The Pearson correlation was also used to determine the associations between self-efficacy and self-care behaviors. Data normality was checked using the Kolmogorov–Smirnov test. The independent *t*-test was used to analyze the 2 groups in terms of the homogeneity of the quantitative variables and the χ^2^ test for the qualitative variables. Repeated measurement design was carried out to investigate the effects of the educational intervention on self-care behaviors. Then, for further investigation, the independent *t*-test was utilized to compare the dimensions of self-care behaviors between the intervention and control groups. The level of significance was set at 0.05. 

## Results

**Figure 1:**
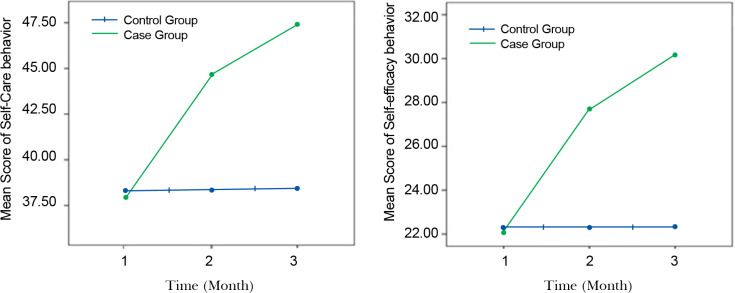
Variation of self-efficacy and self-care behavior across time in both case and control groups after education intervention

In the present study, 80 patients with HF participated. Forty patients were in the control group and 40 in the experimental group. The mean age of the HF patients in the intervention and control groups was 55.00±8.48 and 51.61±8.51 years, respectively. Table 1 shows the sociodemographic characteristics of the studied participants in both groups. The 2 groups were similar in terms of demographic variables. The results of the Pearson correlation test revealed a noticeable positive correlation between self-care and self-efficacy, shortly after the intervention (r=0.82, P<0.001) and 3 months after the intervention (r=0.85, P<0.001). In other words, increased self-efficacy in the HF patients promoted their self-care behaviors. The total mean (±standard deviation) of the patients’ self-efficacy and self-care behavior scores in the experimental and control groups is presented in Table 2. The results of the measurement showed that the educational intervention had a positive effect on self-care and self-efficacy behaviors (Table 2, Figure 1). The mean score of self-efficacy and self-care behaviors in the intervention group was higher than that in the control group after the intervention and at 3 months’ follow-up. Furthermore, over time, the level of the patients’ self-awareness about all the aspects of self-care behaviors rose, thus; the mean score of self-care behaviors in the intervention group increased significantly at 3 months’ follow-up. The results of the independent *t*-test demonstrated that there was no significant difference in the mean score of self-care behaviors and self-efficacy between the 2 groups at the beginning of the study. However, noticeable differences were observed between the intervention and control groups following the intervention and at 3 months’ follow-up (P<0.001) (Table 2). 

**Table 1:** Participants’ demographic information^*^

	Intervention group	Control group	Total	P
Age group (y)				0.471
30-40	6 (15.0)	8 (20.0)	14 (17.5)	
40-59	24 (60.0)	21 (52.5)	45 (56.2)	
60-79	10 (25.0)	11 (27.5)	21 (26.3)	
Gender				0.642
Men	11 (27.5)	10 (0.3)	21 (26.3)	
Women	29 (72.5)	30 (0.7)	59 (73.7)	
Educational level				0.430
High school	25 (62.5)	22 (55.0)	47 (58.8)	
Diploma	11 (27.5)	15 (37.5)	26 (32.5)	
University graduate	4 (10.0)	3 (7.5)	7 (8.7)	
Occupation				0.693
Household	18 (45.0)	20 (50.0)	38 (47.5)	
Free job	13 (32.5)	14 (35.0)	27 (33.7)	
Employed	9 (22.5)	6 (15.0)	15 (18.8)	
Place of residence				1.000
Village	3 (7.5)	2 (5.0)	5 (6.0)	
City	37 (92.5)	38 (95.0)	75 (94.0)	

* Data are presented as n (%)

**Table 2:** Comparison of the self-care dimensions of the patients in the experimental and control groups*

Self-care dimension	Before the intervention	Shortly after the intervention	Three months after the intervention	P in repeated measure analysis
Self-care score**				
Experimental	23.50±6.58	42.62±6.74	48.66±7.02	<0.001
Control	23.53±6.63	23.47±6.73	23.80±7.25	0.069
P value in t-test	0.987	0.014	0.030	
Self-efficacy score***				
Experimental	18.57±6.64	32.29±7.06	36.24±6.59	<0.001
Control	18.55±6.49	18.80±6.17	18.53±7.04	0.059
P value in t-test	0.961	<0.001	<0.001	

* Data are presented as mean±SD

** Total score of this scale ranges from 12 to 60, and lower scores indicate poor observance of self-care behaviors by patients.

*** Total score of the questionnaire is between 0 and 52, and higher scores represent better-perceived self-efficacy.

## Discussion

The current study aimed to determine the efficacy of a training program based on self-efficacy strategies in developing self-care behaviors among patients with HF. The results suggested that educational strategies could be effective in improving patients’ health outcomes insofar as clear communication and the use of understandable written media had a significant impact on the patients’ understanding of self-efficacy and self-care behaviors. Several studies have reported that good self-efficacy is noticeably associated with self-care activities such as controlling diet, adhering to medication, engaging in physical activity, and practicing weight management strategies.^14^^, ^^15^  Individuals with higher self-efficacy can manage most of the resources available to them to master a situation.^   16 ^ In the present study, self-efficacy was improved in the control group, but this difference was not statistically significant. This finding also correlates with the self-efficacy theory, which expresses that the passing of time and the possibility of direct and especially successful experiences increase self-efficacy through performance-enhancing strategies.^           17 ^ The mean of the self-care score in the experimental group was significantly higher at follow-up and after the intervention, indicating that an effective educational intervention was able to play a significant role in improving self-care behaviors in our HF patients. The results were in a good agreement with a study conducted by Barnason et al.,^18^ who showed that educational intervention could be effective in increasing self-care behaviors among patients with chronic HF. Education and promotion of health literacy can play an important role in fostering health and getting the best from the health care system in HF patients. Koelling et al.^19^ reported that hospitalized HF patients who received 1 session of health education before hospital discharge showed a significant reduction in the mortality and readmission rates compared with patients who received only standard written discharge information. The results of the correlation test between self-efficacy and self-care behaviors also exhibited a direct and significant relationship between these 2 variables after the intervention. Therefore, it can be concluded that if a higher increase in the self-efficacy score occurs, the difference in the self-care score will be significantly higher in the intervention group than in the control group, which is consistent with a study done by Chen et al.,^20^ who explained that an increase in self-efficacy would improve self-care behaviors in HF patients. 

Although the findings indicated the effectiveness of the educational intervention, the present study had some limitations including the impact of other information sources on the intervention and control groups and the collection of data via the self-report method, which might have led to an incorrect description of the variables. Further studies that use observational or other highly accurate data collection methods along with the questionnaires are recommended to evaluate the effects of educational training programs on health-related quality of life among HF patients.

## Conclusion

Our findings suggest that heart failure patients with higher self-efficacy are more capable of performing self-care behaviors. The use of self-efficacy strategies can increase patients’ awareness and self-esteem and enable them to self-manage and control their symptoms. Moreover, the use of clear communication techniques, intelligible and illustrated media, and visual teaching aids can improve the health status of patients. Generally, the implementation of effective education strategies for heart failure patients, particularly among those with education less than the high-school level, is of great importance in bolstering their self-efficacy and self-care behaviors. 

## Notes:

***This paper should be cited as: ***Peyman N, Shahedi F, Abdollahi M, Doosti H, Zadehahmad Z. Impact of Self-Efficacy Strategies Education on Self-Care Behaviors among Heart Failure Patients. J Teh Univ Heart Ctr 2020;15(1):6-11.
